# Utility of Calomel in Typhoid Fever

**Published:** 1844-03-23

**Authors:** 


UTILITY OF CALOMEL IN TYPHOID FEVER.

Drs. Lombard and Fauconnet, of Geneva, sum up an elaborate account of their experimental inquiries on this important point of practice in the following words:—

"Calomel diminishes the mortality of typhoid fever, and renders all its symptoms less severe, more especially those that indicate a disturbance of the nervous centres. It tends to abate the danger of any thoracic complications supervening, and, without being able to check them altogether, it causes them to be less serious in their consequences, as well as less frequent in their occurrence. It modifies and corrects in a very remarkable manner the condition of the alvine evacuations, and usually serves to diminish any diarrhœa, if present, and to bring all the secretions to a more normal state. It rapidly cleanses the tongue, and renders it and the mouth less parched; it dissipates tympanitic distention and colicky pains of the bowels, and appears to exercise rather a servicable than an injurious effect on the gastro-intestinal inflammation, which not unfrequently complicates typhoid fever." Drs. L. and F. attribute the efficacy of calomel in this disease to its constitutional operation, rather than to any direct effects which it may have on the abdominal viscera. They remark, that there is almost always a decided amelioration of the symptoms, when the gums become slightly effected with the mercurial irritation.

(The observations of these gentlemen are in accordance with the general experience of most medical men in this country. Mercury seems unquestionably to exercise a very marked, and, in most cases, a very servicable influence on the course of typhoid fever. Its modus operandi is probably twofold; first, by correcting and evacuating the secretions of the bowels; and secondly, by altering and modifying the state of the circulating fluids, as well as the vital powers of the bloodvessels themselves. A favourite preparation with many practitioners is the Hydrargyrum cum cretâ, either alone or in combination with carbonate of soda, ipecacuan, Dover's powder, &c. The use of this mercurial, at stated intervals—in doses of from four to eight grains, every six, eight, or twelve hours—with the exhibition of saline draughts between each dose, is, on the whole, by far the safest medication that can be resorted to in the majority of cases of low fever.)—*London Medico-Chirurgical Review*, Jan., 1844.

